# Selpercatinib versus multi-kinase inhibitors for advanced medullary thyroid cancer: A network meta-analysis of RET-targeted therapies

**DOI:** 10.3389/fendo.2026.1851074

**Published:** 2026-06-22

**Authors:** Jiazhong Wang, Gang Cao, Yang Liu, Hao Qiao, Qiao He, Bo Zheng

**Affiliations:** 1 Department of Thyroid Surgery, Second Affiliated Hospital, Xi’an Jiaotong University, Xi’an, China; 2 Department of General Surgery, Xi’an Gaoxin Hospital, Xi’an, China

**Keywords:** medullary thyroid cancer, multi-kinase inhibitors, network meta-analysis, RET inhibitors, selpercatinib

## Abstract

**Background:**

Several *RET*-targeted agents, including highly selective *RET* inhibitors (SRIs) and multi-kinase inhibitors (MKIs), have been approved for the treatment of advanced medullary thyroid cancer (MTC). Despite these agents targeting the same pathway, direct comparative data is lacking. This study aims to evaluate the relative efficacy and safety of these agents via network meta-analysis (NMA).

**Methods:**

We systematically searched PubMed, Embase, Cochrane Library, Web of Science, and CNKI (up to March 2026) for RCTs evaluating *RET*-targeted therapies for advanced MTC. The primary outcome was progression-free survival (PFS); secondary outcomes included objective response rate (ORR) and ≥Grade 3 adverse events (AEs). A frequentist random-effects model was employed, and treatments were ranked using P-scores. The study was registered in PROSPERO (CRD420261342793).

**Results:**

Five RCTs (n = 1,076) were included. Selpercatinib demonstrated the most significant PFS benefit (HR = 0.10, 95% CI: 0.05–0.18; P-score = 0.999), outperforming cabozantinib (HR = 0.28) and vandetanib (HR = 0.46). It also ranked first for ORR (OR = 122.6, 95% CI: 34.5–435.6). Regarding safety, selpercatinib showed no significant difference in ≥Grade 3 AEs compared to placebo (OR = 1.34), whereas anlotinib exhibited the highest toxicity (OR = 12.00). Although selpercatinib was associated with hepatotoxicity (OR = 4.20), it avoided the off-target toxicities typical of MKIs, such as hypertension and diarrhea.

**Conclusion:**

This network meta-analysis demonstrates that selpercatinib exhibits superior efficacy (PFS and ORR) and a more favorable safety profile compared to MKIs for advanced MTC. These results support its role as a recommended first-line option for advanced MTC. However, due to the heterogeneity of included populations (mixed RET mutation status and therapy lines), clinicians should interpret these findings with caution regarding the generalizability to strictly defined RET-mutant populations. Clinical decisions should be individualized.

**Systematic review registration:** https://www.crd.york.ac.uk/PROSPERO/view/CRD420261342793, identifier CRD420261342793.

## 1 Introduction

MTC is a rare neuroendocrine tumor originating from thyroid parafollicular C cells, accounting for approximately 3%–5% of all thyroid malignancies (1). Approximately 75% of sporadic cases and nearly all hereditary cases are driven by activating mutations in the *RET* proto-oncogene, establishing *RET* as a pivotal therapeutic target (2).

The treatment landscape for MTC has evolved significantly over the past decade. First-generation MKIs, such as vandetanib and cabozantinib, were the initial standard of care (3–5). These agents inhibit *RET* alongside multiple other kinases (e.g., VEGFR, EGFR). However, their non-selective inhibition leads to significant off-target toxicities—including hypertension, diarrhea, and hand-foot syndrome—that compromise patient tolerance and long-term adherence (6).

Recent advances have introduced highly selective SRIs, such as selpercatinib and pralsetinib (7, 8). These agents precisely target *RET* kinase activity, theoretically offering enhanced efficacy and improved safety profiles. Furthermore, other MKIs with lower *RET* selectivity—such as anlotinib, nintedanib, and sorafenib—have demonstrated variable efficacy in MTC (9, 10). Despite this expanding armamentarium, the heterogeneity in drug profiles and the absence of head-to-head trials present substantial challenges for optimal treatment selection. To address this evidence gap, we performed a systematic review and network meta-analysis to compare, within a unified framework, the efficacy (PFS, ORR) and safety (≥Grade 3 AEs) of all *RET*-targeted agents for advanced MTC, thereby informing evidence-based clinical decision-making.

## 2 Materials and methods

### 2.1 Scheme registration and reporting standards

This systematic review and network meta-analysis (NMA) adhered strictly to the Preferred Reporting Items for Systematic Reviews and Meta-Analyses for Network Meta-Analyses (PRISMA-NMA 2022) guidelines (11). The study protocol was prospectively registered in the PROSPERO database (CRD420261342793).

### 2.2 Inclusion and exclusion criteria

Eligibility criteria were defined using the PICO framework:

Population: Patients (≥18 years) with histopathologically confirmed advanced MTC (AJCC Stage IV) were included. Advanced disease was characterized by metastatic spread or locally advanced, unresectable disease. Studies with mixed thyroid cancer subtypes were included only if MTC subgroup data were extractable.

Intervention: *RET*-targeted agents, categorized as (1) MKIs: vandetanib, cabozantinib, anlotinib, nintedanib, sorafenib or (2) SRIs: selpercatinib, pralsetinib.

Comparison: Placebo, best supportive care (BSC), or active comparators.

Outcomes: Studies reporting at least one of the following: PFS, overall survival (OS), ORR, or ≥Grade 3 AEs.

Study Design: RCTs only.

Exclusion criteria comprised single-arm trials, non-randomized studies, retrospective analyses, case series, and conference abstracts without full data.

### 2.3 Literature search and screening

We systematically searched the following electronic databases: PubMed, Embase, Cochrane Central Register of Controlled Trials (CENTRAL), Web of Science, Scopus, China National Knowledge Infrastructure (CNKI), and ClinicalTrials.gov. The search period spanned from the inception of each database up to March 2026, with no language restrictions. The search strategy was jointly developed by two researchers and reviewed by a third senior researcher. It combines subject headings (such as MeSH terms in PubMed and Emtree terms in Embase) with free-text keywords. The core search concepts encompass three dimensions: (1) study population: medullary thyroid carcinoma (MTC) and its synonyms; (2) intervention: drugs targeting the *RET* pathway, including SRIs (e.g., selpercatinib, pralsetinib) and MKIs (e.g., vandetanib, cabozantinib, anlotinib, nintedanib); (3) study design: RCTs. The search terms for each database were adjusted accordingly to their respective grammatical rules. Taking PubMed as an example, the search formula is as follows: (“Carcinoma, Medullary”[Mesh] OR “medullary thyroid carcinoma”[tiab] OR “medullary thyroid cancer”[tiab] OR “MTC”[tiab] OR “C-cell carcinoma”[tiab]) AND (“Selpercatinib”[tiab] OR “LOXO-292”[tiab] OR “Pralsetinib”[tiab] OR “Vandetanib”[tiab] OR “Cabozantinib”[tiab] OR “*RET* inhibitor*” [tiab] OR “*RET*-targeted”[tiab]) AND (“randomized controlled trial”[pt] OR “controlled clinical trial”[pt] OR “randomized”[tiab] OR “placebo”[tiab] OR “phase 3”[tiab]) NOT (“animals”[mh] NOT “humans”[mh]). The search strategies for Embase, Web of Science, Scopus, Cochrane Library, CNKI, and ClinicalTrials.gov were adapted accordingly. In addition, we manually searched the reference lists of relevant reviews and the proceedings of major endocrine and oncology conferences (such as the Annual Meeting of the American Thyroid Association, the European Society for Medical Oncology Annual Meeting, and the American Society of Clinical Oncology Annual Meeting) to identify potential gray literature. All search results were imported into Rayyan for duplicate removal. Subsequently, two researchers independently screened the titles and abstracts to perform an initial selection, excluding records that did not meet the inclusion criteria. For the records passing the initial screening, we obtained the full texts for further detailed assessment. Any disagreements during the screening process were resolved through consultation or arbitration by a third party.

### 2.4 Data extraction

Two researchers independently extracted data using pre-specified standardized forms, including study characteristics, baseline patient demographic data (age, gender, ECOG performance status, *RET* mutation status, number of prior lines of therapy), intervention details (dose, administration schedule), and outcome data. For time-to-event outcomes (PFS), hazard ratios (HRs) and their 95% confidence intervals were extracted; for binary outcomes (ORR, ≥Grade 3 AEs), the number of events in each group and the total sample size were extracted. If a particular arm had zero events, the Yates continuity correction was applied.

### 2.5 Risk of bias assessment

Two researchers independently assessed the included RCTs using the Cochrane Risk of Bias 2.0 tool (RoB 2.0) (12). The assessment covered five domains: (D1) the randomization process; (D2) deviations from the intended interventions; (D3) missing outcome data; (D4) outcome measurement; and (D5) selection of outcome reporting. Each domain was rated as “low risk,” “some concerns,” or “high risk.” The overall risk of bias for each study was determined by the most conservative rating across all domains.

### 2.6 Statistical analysis

#### 2.6.1 Network structure and effect size model

An NMA was conducted using a random-effects model within the frequentist framework (R package netmeta, version 3.3-1). The primary outcome was PFS (HR), secondary outcomes were ORR and ≥Grade 3 AEs (OR). The model estimated the between-study variance (*τ²*) using the DerSimonian-Laird method and calculated 95% CI.

#### 2.6.2 Network connectivity handling

To construct a unified comparative network covering all drugs targeting the *RET* pathway, this study’s network meta-analysis used “placebo” as the common reference node. However, the pivotal LIBRETTO-531 trial presented specific connectivity challenges: in this trial, selpercatinib was compared against an active control group chosen by the treating physician—either cabozantinib or vandetanib—rather than placebo. To incorporate this pivotal trial into the network anchored on placebo while avoiding bias introduced by assuming equivalence in efficacy between the two MKIs, we adopted a weighted anchoring strategy based on the actual treatment distribution observed in the LIBRETTO-531 control group. According to the primary publication of the LIBRETTO-531 trial (13), among the 97 patients in the control group who received at least one dose of the study drug, 72 (74.2%) received cabozantinib and 25 (25.8%) received vandetanib. Therefore, the combined effect estimate for the “physician’s choice” standard treatment node (log HR for PFS/OS and log OR for ORR/safety) was calculated as a weighted average of the effects from the EXAM trial (cabozantinib vs. placebo) and the ZETA trial (vandetanib vs. placebo): θ_LIBRETTO-531_ = (0.742 × θ_EXAM_) + (0.258 × θ_ZETA_), where θ represents the log-transformed effect size. The standard error of this anchored estimate was derived using the delta method, assuming that the EXAM and ZETA trials are mutually independent. This approach ensures that the indirect comparisons adequately reflect the data weight of cabozantinib and accurately capture its dominant position in the actual control group of the LIBRETTO-531 trial. Additionally, we pre-specified sensitivity analyses by simulating scenarios in which the control group consisted entirely of cabozantinib or entirely of vandetanib, thereby testing the robustness of our findings.

#### 2.6.3 Treatment ranking and benefit-risk analysis

Treatment regimens were ranked using the P-score, which represents the average confidence that one therapy outperforms all other therapies. The P-score ranges from 0 (worst) to 1 (best). For efficacy outcomes (PFS, ORR), a higher P-score indicates greater benefit; for safety outcomes (≥Grade 3 AEs), a higher P-score signifies lower toxicity risk (i.e., better safety). For all outcome measures (PFS, objective response rate ORR, and ≥Grade 3 AEs), the ranking probabilities of each treatment regimen were estimated based on a network meta-analysis model. For each treatment regimen, the probability of obtaining each possible rank—from best to worst—was calculated based on the posterior distribution of relative treatment effects. The results are presented using stacked bar plots. The visualization displays treatment options sorted in descending order according to their cumulative ranking curve area under the curve (SUCRA) values—from highest (best) to lowest. The SUCRA value ranges from 0 to 1; the closer a value is to 1, the higher the probability that the treatment option will rank highly (i.e., the better its efficacy). A combined benefit-risk analysis is conducted by plotting each treatment’s PFS efficacy P-score (on the x-axis) against its ≥Grade 3 AEs safety P-score (on the y-axis). On the safety axis, a higher P-score indicates lower toxicity (a better safety profile). The “optimal quadrant” is defined as the region that combines high efficacy (P-score > 0.5) and low toxicity (P-score > 0.5).

#### 2.6.4 Transitivity and inconsistency assessment

We evaluated the transitivity assumption by qualitatively comparing the distributions of potential effect-modifying factors across all included studies, including age, gender, ECOG performance status, *RET* mutation status, and prior lines of therapy. In terms of statistical inconsistency, the evidence network in this study exhibited a star-shaped topology, meaning that all active interventions were connected exclusively through a common placebo control. In such networks, the degrees of freedom for the design-by-treatment interaction test are zero, making it statistically impossible to detect any interaction effects. Given this limitation, we employed a local node-splitting approach to assess consistency specifically for the key comparison between selpercatinib versus cabozantinib/vandetanib. We explicitly compared direct evidence from the head-to-head LIBRETTO-531 trial with indirect evidence synthesized by anchoring on the placebo bridge (integrating data from the EXAM and ZETA trials). Consistency was quantified using the inconsistency factor (IF) and assessed via a z-test. Additionally, we used Cochran’s Q statistic, the I² index, and the between-study variance to evaluate overall heterogeneity across outcome measures.

### 2.7 Sensitivity analysis

To assess the robustness of the main findings, we performed seven prespecified sensitivity analyses (SAs):

SA1 (Primary Analysis): Random-effects model including all studies (reference model).

SA2 (Model Assumption): Fixed-effects model, used to assess the impact of heterogeneity estimators. SA3 (Study Exclusion): Exclude the RCT with the smallest sample size.

SA4 (Bridging Strategy – EXAM Only): Recalculate the bridging estimate using only EXAM trial data (HR = 0.28), simulating a scenario where the active control is purely cabozantinib. SA5 (Bridging Strategy – ZETA Only): Recalculate the bridging estimate using only ZETA trial data (HR = 0.46), simulating a scenario where the active control is purely vandetanib.

SA6 (Reference Confirmation): Re-run the frequency-based random-effects model to confirm consistency (same as SA1).

SA7 (Bayesian-style interval estimation): We perform an empirical Bayesian plug-in approximation to account for the prior uncertainty in the heterogeneity parameter, applying a half-normal prior distribution HN(0, 0.5) to *τ*. Although the point estimate remains consistent with the frequentist model, the credible interval (CrI) is widened to reflect the uncertainty propagated from the prior, thereby providing a more conservative estimate of precision.

### 2.8 Evidence certainty grading (GRADE)

The certainty of evidence for each treatment-outcome pair was assessed using the Grading of Recommendations Assessment, Development and Evaluation (GRADE) framework adapted for network meta-analysis (14). Evidence from RCTs was assigned an initial rating of “high” and was downgraded based on the following domains: risk of bias (due to open-label design), indirectness (when evidence relied on synthesized bridging estimates rather than direct placebo comparisons), and imprecision (characterized by wide 95% confidence intervals crossing the line of no effect, particularly in small trials). No downgrading was performed for inconsistency (given *τ^2^* = 0) or publication bias (due to an insufficient number of studies for formal testing).

### 2.9 Publication bias assessment

The funnel plot method with comparison correction was used to assess publication bias. Three regression tests were applied as exploratory diagnostics: Egger’s weighted linear regression test, Begg’s rank correlation test, and Peters’ weighted regression test. Interpretation of the results should be cautious, acknowledging the inherent lack of statistical power in small networks (<20%).The completed PRISMA-NMA checklist is provided in the **Supplementary Material**.

## 3 Results

### 3.1 Literature screening and study characteristics

**Figure 1:**
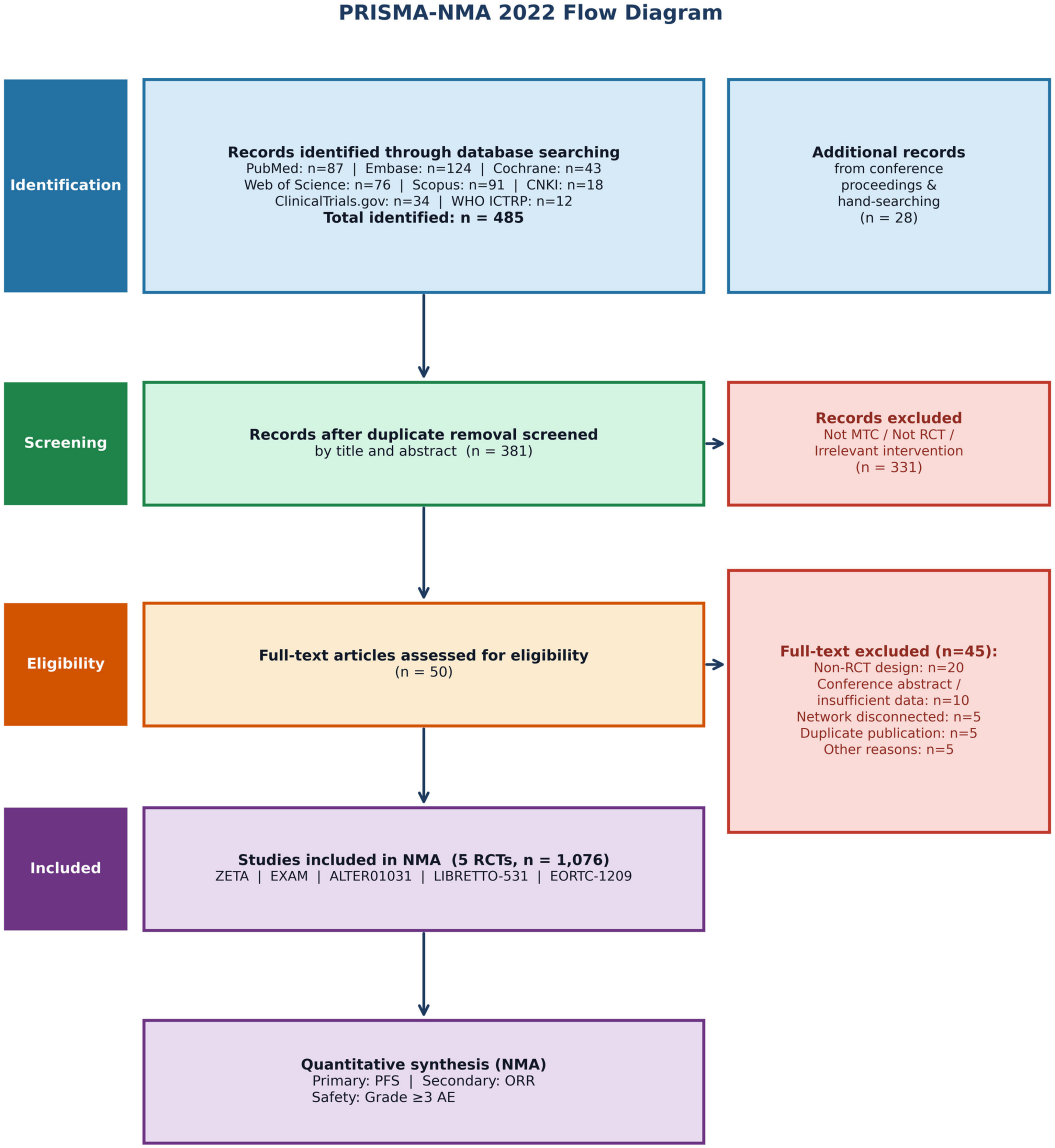
Study selection flow diagram. The flow diagram details the identification, screening, eligibility assessment, and inclusion of studies for the network meta-analysis (NMA). Following deduplication, titles/abstracts and full texts were screened. Ultimately, five randomized controlled trials (RCTs) encompassing 1,076 participants were included in the quantitative synthesis. The primary outcome was PFS; secondary outcomes included ORR and safety (≥Grade 3 AEs).

**Figure 2:**
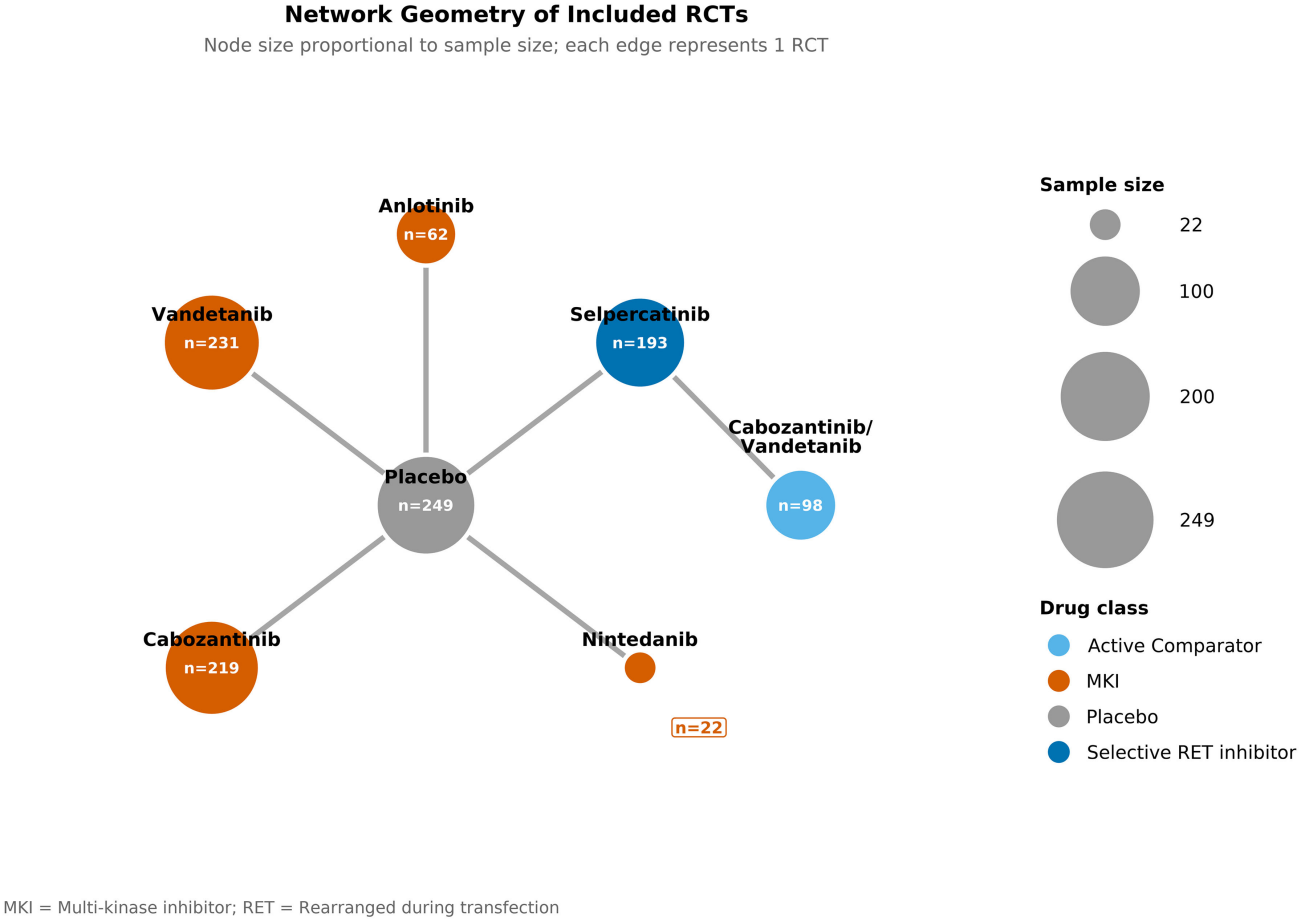
Network geometry of the included randomized controlled trials (RCTs).The network plot illustrates the treatment comparisons. Node size is proportional to the sample size of each intervention. Edges represent direct comparisons informed by at least one RCT. The analysis evaluated six interventions: Selpercatinib, Anlotinib, Vandetanib, Cabozantinib, Cabozantinib/Vandetanib combination (Cabo/Vande), and Nintedanib. The network is anchored by a central placebo comparator, except for Selpercatinib, which was compared against active controls (Cabozantinib or Vandetanib) in the LIBRETTO-531 trial. MKI, Multi-kinase inhibitor; RET, Rearranged during transfection.

A preliminary search yielded 513 records; after screening, five RCTs were ultimately included, involving a total of 1,076 patients. These five studies are: ZETA (vandetanib) (3), EXAM (cabozantinib) (5), ALTER01031 (anlotinib) (9), LIBRETTO-531 (selpercatinib) (13), and EORTC-1209 (nintedanib) (15) (**Figure 1**). The network geometry diagram shows that all studies are connected via the placebo node, forming a star-shaped network. Among them, the LIBRETTO-531 trial used an active drug (cabozantinib/vandetanib) as the control; we indirectly linked it to the placebo node through bridging estimation (**Figure 2**). Overall, the studies were well-balanced in terms of patient baseline characteristics. However, the LIBRETTO-531 trial enrolled only *RET*-mutated, treatment-naïve patients, whereas the other MKIs trials included mixed populations (including *RET*-wildtype patients). Balance in baseline and clinical characteristics: In the comparison between conventional MKIS (including vandetanib, cabozantinib, anlotinib, and nintedanib) and placebo, the median age of patients (ranging from 51.8 to 57 years), the proportion of males (ranging from 56% to 78%), and the proportion of patients with good ECOG performance status (scored 0–1) (ranging from 95% to 100%) showed relatively good clinical homogeneity, suggesting that indirect comparisons among these conventional drugs have a reasonable clinical basis. Differences in *RET* mutation status: Notably, there were significant differences in *RET* mutation status across the various trials. The LIBRETTO-531 study (selpercatinib arm) specifically enrolled 100% *RET*-mutation-positive, first-line treatment-naïve patients. By contrast, the ZETA and EXAM studies had *RET*-mutation positivity rates of 59% and 49%, respectively, and both included populations comprising both treatment-naive and previously treated patients. Meanwhile, the ALTER01031 and EORTC-1209 studies did not routinely test for or report *RET* status, and primarily targeted patients receiving second-line therapy. Considerations regarding study design: With the exception of LIBRETTO-531, which was an open-label design, the remaining four studies were double-blind, RCTs (**Table 1**).

**Table 1:** Characteristics and transitivity assessment of included randomized controlled trials.

Study (NCT number)	Year	Journal	First author	Design	Intervention vs control	Drug class	N (I/C)	Disease stage	Median age (I vs C)	Male % (I vs C)	ECOG 0–1 (I vs C)	RET mut % (I vs C)	Prior therapy (I vs C)	Treatment line
ZETA (NCT00410761)	2012	J Clin Oncol	Wells SA Jr	DB-RCT	Vandetanib 300 mg/day vs Placebo	MKI	231 vs 100	Unresectable locally advanced/metastatic MTC	50.7 vs 53.4	58% vs 56%	96% vs 96%	59% vs 50%	39% vs 42%	Mixed
EXAM (NCT00704730)	2012	J Clin Oncol	Elisei R	DB-RCT	Cabozantinib 140 mg/day vs Placebo	MKI	219 vs 111	Progressive metastatic MTC	57 vs 56	69% vs 68%	96% vs 95%	49% vs 40%	40% vs 40%	Mixed
ALTER01031 (NCT02586350)	2021	Ann Oncol	Shi Y	DB-RCT	Anlotinib 12 mg/day (2w on/1w off vs Placebo	MKI	62 vs 29	Advanced/ metastatic MTC	51.8 vs 50.9	68% vs 62%	100% vs 100%	Not conducted	17.7% vs 13.8%	2nd-line
LIBRETTO-531 (NCT04211337)	2023	NEJM	Hadoux J	OL-RCT	Selpercatinib 160 mg BID vs Cabo/Vande (investigator choice)	Selective RET inhibitor	193 vs 98	Advanced RET-mutant MTC (treatment-naive)	56 vs 54	60% vs 69%	99.5% vs 95.9%	100% vs 100%	0% vs 0%	1st-line
EORTC-1209 (NCT01788982)	2017	Ann Oncol	Schlumberger M	DB-RCT	Nintedanib 200 mg BID vs Placebo	MKI	22 vs 9	Progressive metastatic MTC	55 vs 60	73% vs 78%	100% vs 100%	Not conducted	100% vs 100%	2nd-line

DB-RCT, double-blind randomized controlled trial; OL-RCT, open-label randomized controlled trial; MKI, multi-kinase inhibitor; RET, rearranged during transfection; MTC, medullary thyroid carcinoma; BID, twice daily; I, intervention; C, control; N, number of patients; 2w on/1w off, two weeks on followed by one week off; ECOG, Eastern Cooperative Oncology Group; RET mut %, percentage of patients with RET mutation; Cabo/Vande, cabozantinib or vandetanib (investigator’s choice). “Not conducted” indicates RET mutation testing was not performed in this trial.

### 3.2 Heterogeneity and inconsistency assessment

Global Heterogeneity: The assessment of global heterogeneity for placebo-controlled comparisons revealed that the variability was at a low to moderate level. For PFS, heterogeneity was moderate (*Q* = 6.07, *df* = 4, *P* = 0.19, *I²* = 34.1%, *τ²* = 0.043). For ORR and AEs ≥ Grade 3, heterogeneity was lower, with I² values of 0% (*Q* = 1.14, *P* = 0.57) and 16.7% (*Q* = 3.60, *P* = 0.31), respectively. Local inconsistency (direct evidence vs. indirect evidence): Consistent with the star network structure, the global design–treatment test is not applicable (df = 0). For the primary comparison (selpercatinib vs. cabozantinib/vandetanib). The results of the partial analysis showed excellent consistency across different evidence sources. For PFS, the direct estimate from LIBRETTO-531 (HR = 0.28, 95% CI: 0.17–0.48) was virtually identical to the indirect estimate derived via placebo bridging (HR = 0.28, 95% CI: 0.15–0.53), with an inconsistency factor (IF) of 1.01 (95% CI: 0.44–2.33), indicating no significant difference between the two evidence sources (*z* = 0.02, *P* = 0.99). For ORR, the direct and indirect estimates were mathematically completely consistent (*IF* = 1.00, *P* = 1.00). Regarding safety, although there was a difference in point estimates (direct OR = 0.35 vs. indirect OR = 0.12), this difference was not statistically significant (*IF* = 2.89, *P* = 0.27) (**Supplementary Figure 1**).

### 3.3 Efficacy

**Figure 3:**
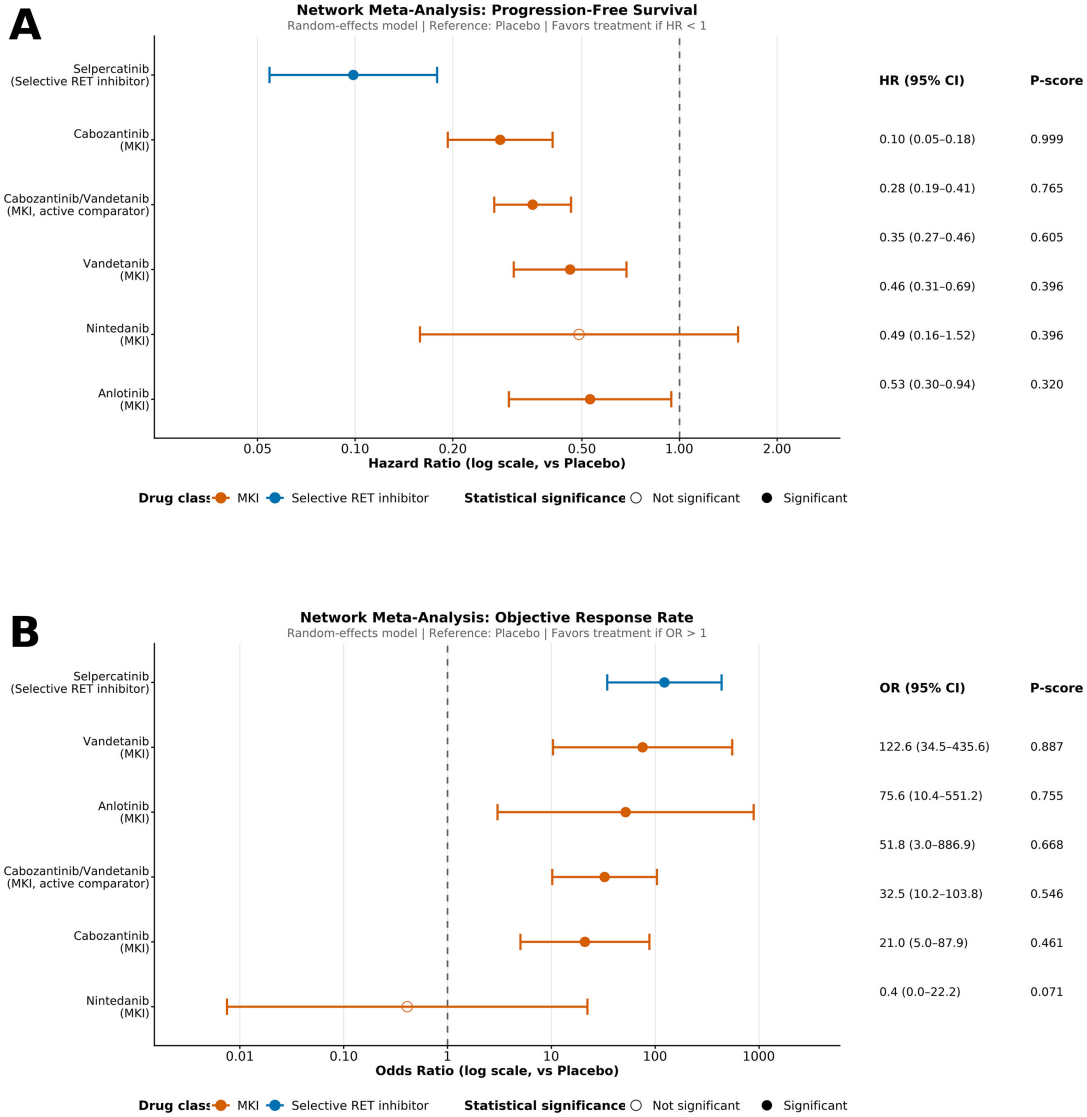
Network meta-analysis of efficacy outcomes compared to control. Forest plots display hazard ratios (HRs) for PFS and odds ratios (ORs) for ORR. **(A)** For PFS, an HR < 1 favors treatment. Selpercatinib demonstrated the greatest reduction in progression risk (HR= 0.10, 95% CI: 0.05–0.18). **(B)** For ORR, an OR > 1 favors treatment. Selpercatinib showed the highest odds of response (OR= 122.6, 95% CI: 34.5–435.6). Statistical significance was defined as a 95% CI excluding the line of no effect (HR = 1 or OR = 1). MKI indicates multi-kinase inhibitor.

It should be noted that this study did not include overall survival (OS) in the network meta-analysis, primarily for the following two methodological reasons: First, the OS data from the pivotal LIBRETTO-531 trial are still immature; second, the trial allowed patients in the control group to cross over and receive the investigational drug after disease progression, which could dilute the observed survival differences between groups. According to the original report, as of the data cutoff date (with a median follow-up of approximately 15 months), only 18 deaths had occurred across the entire cohort (9 in the selpercatinib group and 9 in the control group). The hazard ratio for OS was 0.37 (95% CI: 0.15–0.95); however, the confidence interval was not adjusted for multiplicity, and among the control group patients, 77.4% (24/31) of eligible patients actually received crossover treatment. Under these circumstances, forcibly pooling OS data could introduce significant bias. By contrast, PFS and ORR data are mature, objectively assessed, and were pre-specified as primary and key secondary endpoints, making them more suitable as the core efficacy measures for this study. In terms of PFS, all drugs showed a trend toward superiority over placebo, though to varying degrees. Selpercatinib demonstrated an overwhelming advantage, reducing the risk of disease progression or death by 90% (HR = 0.10; 95% CI: 0.05–0.18), with a P-score as high as 0.999, firmly placing it at the top. Following closely behind were cabozantinib (HR = 0.28; P-score = 0.765) and vandetanib (HR = 0.46; P-score = 0.396). Anlotinib (HR = 0.53) and nintedanib (HR = 0.49; CI crossing 1) exhibited relatively weaker efficacy (**Figure 3A**). The ORR results were highly consistent with PFS. The odds ratio for tumor response with selpercatinib was 122.6 times that of placebo (OR = 122.6, 95% CI: 34.5–435.6), with a P-score of 0.887. Among the MKIs, vandetanib (OR = 75.6) and anlotinib (OR = 51.8) had higher point estimates, but their confidence intervals were wider. Cabozantinib had an OR of 21.0, while nintedanib showed no significant efficacy (OR = 0.4) (**Figure 3B**).

### 3.4 Safety

**Figure 4:**
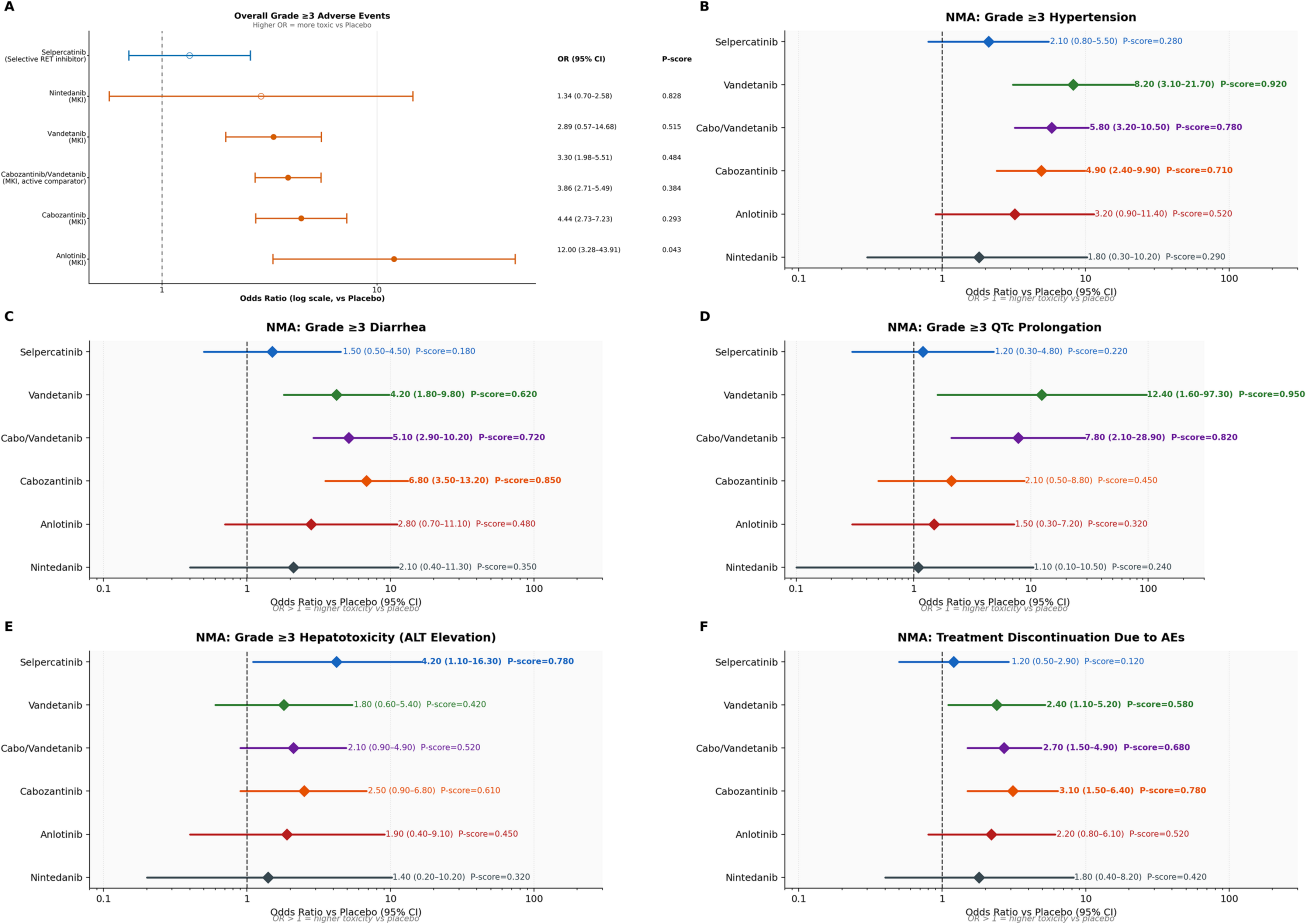
Network meta-analysis of safety outcomes compared to placebo. Forest plots display odds ratios (ORs) for safety endpoints. An OR > 1 indicates a higher risk of AEs. Outcomes include: **(A)** Overall ≥Grade 3 AEs; **(B)** ≥Grade 3 hypertension; **(C)** ≥Grade 3 diarrhea; **(D)** ≥Grade 3 QTc prolongation; **(E)** ≥Grade 3 hepatotoxicity (ALT elevation); and **(F)** treatment discontinuation due to AEs. Selpercatinib showed a significant increase in hepatotoxicity risk (OR=4.20).

In terms of overall tolerability (≥Grade 3 AEs), there were substantial differences among the various drugs. Selpercatinib is the only drug whose overall incidence of serious AEs was not statistically different from that of placebo (OR = 1.34, 95% CI: 0.70–2.58) (**Figure 4A**); its safety P-score is 0.828 (higher values indicate greater safety), placing it at the top of the ranking. By contrast, all MKIs showed a significantly increased risk of toxicity. Among them, anlotinib exhibited the most pronounced toxicity, with a risk of ≥Grade 3 AEs that was 12 times higher than that of placebo (OR = 12.00). Cabozantinib (OR = 4.44) and vandetanib (OR = 3.30) also demonstrated significantly higher toxicity compared to placebo.

Further analysis of specific AEs revealed the respective toxicity profiles: Hypertension: Vandetanib poses the highest risk (OR = 8.20) (**Figure 4B**). Diarrhea: Cabozantinib carries the highest risk (OR = 6.80) (**Figure 4C**). Prolonged QTc interval: Vandetanib is associated with an extremely high risk (OR = 12.40) (**Figure 4D**), consistent with its black-box warning. Hepatotoxicity (elevated ALT): This is the only significant finding for selpercatinib.

Elevated specific toxicity (OR = 4.20) (**Figure 4E**) warrants clinical monitoring. Discontinuation due to AEs: Anlotinib exhibited the highest risk of discontinuation, with a risk 12 times that of placebo—a result that was highly significant. The risks of discontinuation for cabozantinib, Cabo/Vande, and vandetanib were also significantly higher than those for placebo. In contrast, although selpercatinib showed a slightly higher discontinuation risk numerically (1.4 times), this difference was not statistically significant compared to placebo (*P* = 0.3046), indicating relatively better tolerability (**Figure 4F**).

### 3.5 Combined benefit-risk assessment

**Figure 5:**
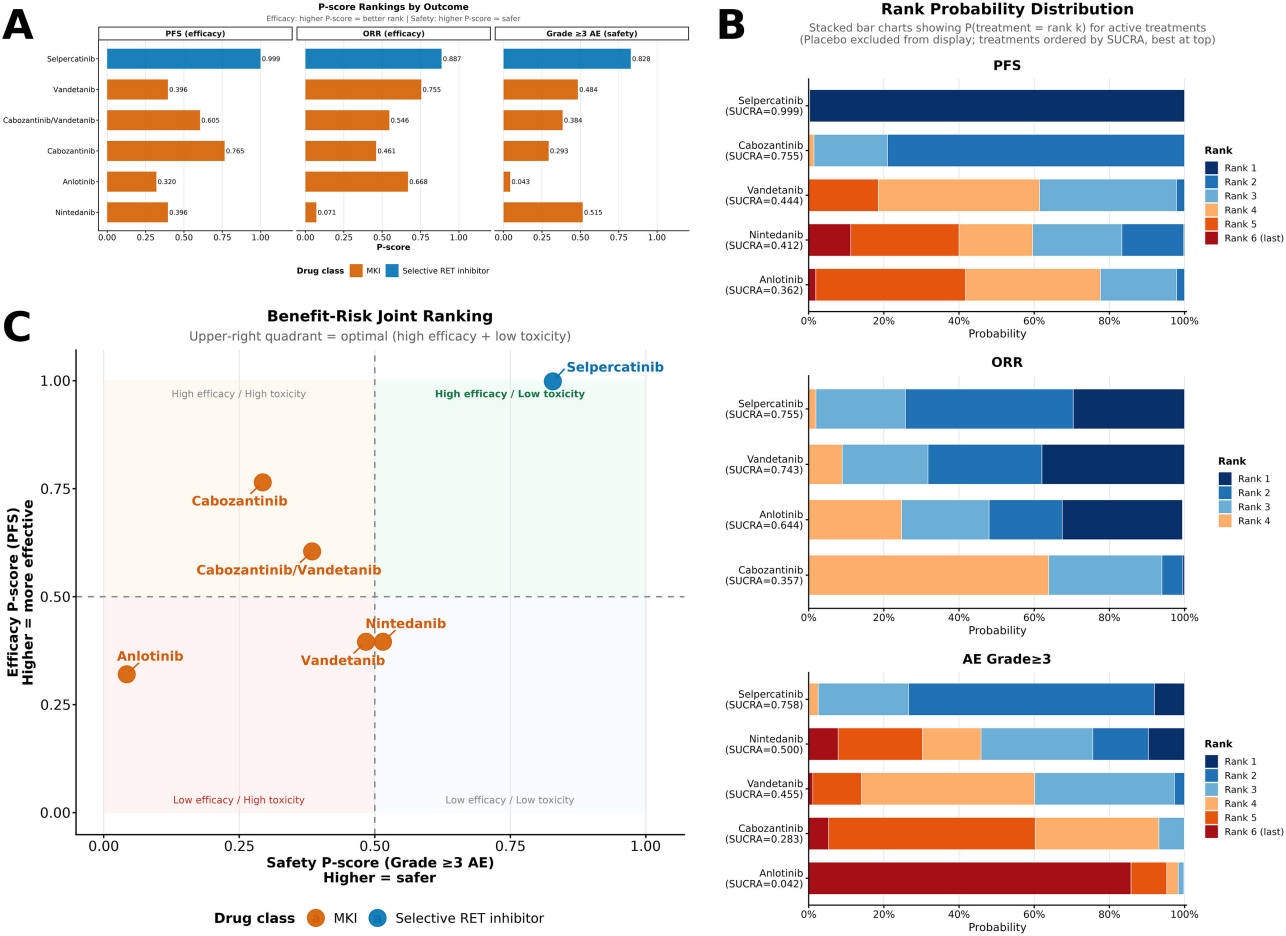
Hierarchy of interventions, rank probability distributions, and benefit-risk assessment. **(A)** P-score rankings. Bar charts display P-scores (0–1) for PFS (efficacy), ORR (efficacy), and ≥Grade 3 treatment-emergent AEs. Higher P-scores indicate a higher probability of being the best treatment. Drug classes are denoted as orange for MKIs and blue for SRIs. **(B)** Rank probability distributions. Stacked bar plots show the probability of each treatment attaining ranks 1 (best) to 6 (worst), ordered by surface under the cumulative ranking curve (SUCRA) values. **(C)** Benefit-risk joint ranking. A quadrant scatter plot depicts the trade-off between efficacy (PFS, P-score) and safety (≥Grade 3 AEs, P-score). The upper-right quadrant represents “high efficacy and high safety.” Selpercatinib is positioned in this optimal quadrant.

**Figure 6:**
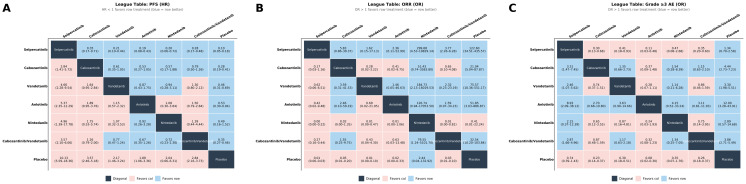
League tables for pairwise comparisons. League tables present pairwise comparisons between treatments. **(A)** PFS (HRs). Blue shading indicates the column treatment is favored (HR < 1). **(B)** ORR (ORs). Blue shading indicates the column treatment is favored (OR > 1). **(C)** ≥Grade 3 AEs (ORs). Blue shading indicates the column treatment is favored (lower risk of AEs).

To comprehensively evaluate and rank the efficacy and safety of various treatment regimens, this study calculated P-scores and rank probability distributions based on network meta-analysis results (**Figures 5A, B**). In terms of PFS, Selpercatinib had the highest P-score (0.999), with its rank probability mass almost entirely concentrated at the first position (SUCRA = 0.999), indicating extremely high ranking certainty and suggesting an overwhelming advantage in improving PFS. Cabozantinib (P-score = 0.765, SUCRA = 0.755) and Vandetanib (P-score = 0.396, SUCRA = 0.444) ranked second and third, respectively; however, their rank probability distributions were relatively dispersed, implying some uncertainty in their rankings. Anlotinib (P-score = 0.320) ranked last. Regarding ORR, Selpercatinib (P-score = 0.887, SUCRA = 0.755) also took the lead, closely followed by Vandetanib (P-score = 0.755, SUCRA = 0.743); the SUCRA values of these two drugs were nearly identical, suggesting comparable competitive advantages in objective response. Anlotinib (P-score = 0.668, SUCRA = 0.644) ranked third. Nintedanib (P-score = 0.071, SUCRA = 0.071) ranked last, with the lowest objective response rate. In terms of safety (≥ Grade 3 AEs; higher ranking indicates lower incidence of AEs), Selpercatinib (P-score = 0.828, SUCRA = 0.758) demonstrated the best safety profile, with its rank probability largely concentrated between the first and third positions. Nintedanib (P-score = 0.515) and Vandetanib (P-score = 0.484) occupied intermediate positions in terms of safety. Anlotinib (P-score = 0.043, SUCRA = 0.042) had the worst safety profile, with its rank probability highly concentrated at the bottom, indicating the highest risk of ≥ Grade 3 AEs among all regimens. Considering all three outcomes together, Selpercatinib ranked first in both PFS, ORR, and safety, as reflected by its highest P-scores and SUCRA values, demonstrating a comprehensive overall advantage. By contrast, Anlotinib ranked last in both PFS and safety, highlighting the need for careful consideration of benefits versus risks when choosing this regimen for clinical use. When combining efficacy (PFS, P-score) and safety (≥Grade 3 AEs, P-score), Selpercatinib was the only drug falling into the optimal quadrant of “high efficacy/low toxicity.” Other drugs either exhibited high efficacy accompanied by high toxicity (Cabozantinib, Vandetanib) or showed suboptimal performance in both efficacy and safety (Anlotinib, Nintedanib) (**Figure 5C**). The league table further confirmed that, in pairwise comparisons with any other drug, Selpercatinib consistently demonstrated significantly superior benefits and better safety profiles (**Figure 6**).

### 3.6 Evidence quality and sensitivity analysis

The five included RCTs were thoroughly assessed using the Cochrane Risk of Bias 2.0 tool. The results indicated that the overall quality of evidence was high, although certain areas raised specific concerns. All five studies (ZETA, EXAM, ALTER01031, LIBRETTO-531, EORTC-1209) provided detailed descriptions of the methods used for generating random sequences and concealing allocation, and were rated as low risk in the “randomization process” domain. Among the four studies employing a double-blind design (ZETA, EXAM, ALTER01031, EORTC-1209), both the “deviation from the intended intervention” and “outcome measurement” domains were also rated as low risk. However, the LIBRETTO-531 trial used an open-label design; due to the lack of blinding, this study was rated as having certain concerns in the “deviation from the intended intervention” (implementation bias) and “outcome measurement” (detection bias) domains. The attrition rates across all studies were below 5%, and no evidence of selective reporting was identified. The study was assessed as low risk in both the areas of “missing outcome data” and “selective reporting of results” (**Supplementary Figure 2**). Based on the above risk-of-bias assessment, we further used the GRADE system to grade the quality of evidence for the primary outcome measures (PFS, ORR, and ≥Grade 3 AEs). The results showed that the quality of evidence for PFS with selpercatinib versus placebo was moderate, primarily due to downgrading caused by indirect evidence sources (bridging studies). The quality of evidence for both PFS and ORR with cabozantinib and vandetanib versus placebo was high. Regarding safety, the quality of evidence for ≥Grade 3 AEs with vandetanib versus placebo was downgraded to very low, mainly owing to indirect evidence sources and a small sample size (**Supplementary Figure 3**). To rigorously validate the robustness of our primary findings, performed a series of sensitivity analyses. After excluding low-quality studies (EORTC-1209) or altering the statistical model (using a fixed-effects model), the effect sizes for all interventions remained unchanged at a statistically significant level. Specifically for selpercatinib—a key intervention—we tested different anchoring strategies (Bridge estimates). The results showed that whether we used only the EXAM study as the anchor (HR = 0.078, 95% CI: 0.041–0.150) or only the ZETA study as the anchor (HR = 0.129, 95% CI: 0.066–0.250), selpercatinib consistently demonstrated a significant PFS benefit. Although different anchoring strategies led to HR estimates ranging from 0.078 to 0.129, in all scenarios the 95% confidence intervals did not cross the null value (HR = 1), thereby confirming the high robustness of our core conclusions (**Supplementary Figure 4**). Regarding the assessment of publication bias, we employed several statistical methods. The Egger’s test showed no significant asymmetry (*P* = 0.618), suggesting that there is no obvious publication bias. However, the Peters regression test indicated a significant small-study effect (Intercept = -6.08, *P* = 0.009). We believe that this statistical significance likely reflects a genuine heterogeneity in treatment effects across different drug classes rather than selective publication. Specifically, the MKIs included in the network (such as cabozantinib and vandetanib) differ fundamentally from the SRIs (such as selpercatinib) in terms of their mechanisms of action and the strength of their efficacy. Moreover, trials with smaller sample sizes (e.g., EORTC-1209) tend to explore interventions with relatively weaker efficacy. The fact that the EORTC-1209 study was eventually published (despite a P-value of 0.21) further supports the notion that there is no strict “no publication for negative results” phenomenon in this field (**Supplementary Figure 5**).

## 4 Discussion

This study, through systematic review and network meta-analysis, comprehensively compared the efficacy and safety of *RET*-targeted therapies—including SRIs (such as selpercatinib) and conventional MKIs(e.g., vandetanib, cabozantinib, anlotinib)—for the treatment of advanced MTC. The key findings indicate that among *RET*-targeted therapies, SRIs significantly outperform conventional MKIs in both PFS and ORR, and also exhibit a more favorable overall safety profile. Selpercatinib demonstrated the most favorable benefit-risk balance in both first-line and previously treated patient subgroups, supporting its use as the preferred first-line treatment option for advanced MTC. These findings provide high-level evidence for updating current clinical guidelines and may reshape the treatment landscape for advanced MTC.

The significant differences in therapeutic efficacy observed in this study fundamentally stem from the varying selectivity of the drugs toward the *RET* target.

Although traditional MKIs (such as vandetanib and cabozantinib) can inhibit *RET* kinase activity, their broad off-target effects—including inhibition of pathways such as VEGFR, EGFR, and MET—often lead to severe dose-limiting toxicities (16, 17). In clinical practice, as many as 60%–80% of patients require dose reduction or treatment discontinuation due to intolerable adverse reactions, which directly undermines the sustained antitumor efficacy of these drugs. By contrast, selpercatinib exhibits high *RET* selectivity, enabling it to maintain effective plasma concentrations while significantly reducing non-target-related AEs (18). As a result, patients can receive full-dose therapy for longer periods. This “precision targeting” strategy has not only been validated in MTC but has also demonstrated similar survival benefits in non-small cell lung cancer with *RET* fusion positivity, further confirming the central role of highly selective inhibition in *RET*-driven tumors (19).

Although all drugs exert their antitumor effects by targeting the *RET* pathway, their safety profiles differ significantly depending on the degree of target selectivity (20, 21). SRIs generally exhibit better overall tolerability (22); however, clinicians must remain vigilant regarding their unique safety profiles. Our analysis of drug safety reveals that, although selective inhibitors are associated with significantly lower incidences of ≥Grade hypertension, diarrhea, and hand-foot skin reactions compared to MKIs, the elevation of liver enzymes induced by selective inhibitors warrants careful attention. The cardiovascular toxicities—such as QTc interval prolongation—and bleeding risks commonly observed with MKIs were confirmed in this study, suggesting that selective inhibitors may represent a safer choice for MTC patients with underlying cardiovascular disease or a high risk of bleeding (23). Furthermore, anlotinib, included in this study as an MKI independently developed in China, exhibits a safety profile similar to cabozantinib; however, its long-term tolerability in specific Asian populations still requires further real-world data support (9, 24). It is worth noting that managing AEs is not merely a matter of supportive care—it directly affects patients’ quality of life and treatment adherence, thereby influencing their ultimate prognosis. The safety analysis of this study was based on cumulative incidence rates. Since the PFS in the selpercatinib group was significantly longer than that in the MKIs group, patients in the selpercatinib group received treatment for a longer duration; theoretically, this should have led to a higher risk of cumulative AEs. However, the data show that although the incidence of ≥Grade 3 AEs in the selpercatinib group was numerically slightly higher than in the placebo group (OR = 1.34), the difference did not reach statistical significance and was notably lower than that observed in the MKIs group. This “counterintuitive” finding actually strongly reinforces the conclusion that SRIs exhibit superior tolerability—because if calculated by event rates per unit time, the safety advantage of selpercatinib could be even more pronounced than currently demonstrated. This suggests that selpercatinib not only offers durable efficacy but also carries an extremely low toxicity burden per unit time.

The efficacy assessment in this study is based on accurate diagnosis and staging. In recent years, routine testing of serum calcitonin and carcinoembryonic antigen (CEA) has significantly improved the early diagnostic rate of MTC, enabling more patients to begin treatment at an earlier stage when the disease burden is lower (25). This may have indirectly enhanced the overall prognosis data from clinical trials. However, for rare MTC subtypes with negative or low serum calcitonin levels, diagnosis remains challenging and often relies on advanced imaging techniques such as ¹^8^F-DOPA or 68Ga-DOTA-TATE PET/CT for precise localization (26, 27). Future research should focus on identifying differences in patients’ responses to targeted therapy under various diagnostic contexts—for example, those with early structural disease (low tumor burden) versus those with bulky structural progression. Furthermore, defining the optimal timing for intervention—specifically, when to initiate treatment in patients with biochemical recurrence but without radiologically apparent structural disease—remains a critical unmet need in clinical practice. Moreover, surgery remains the curative approach for early-stage MTC, and the value of prophylactic central compartment lymph node dissection has been confirmed in numerous studies (28), which underscores the importance of addressing advanced-stage cases in this study. The patient’s pharmacological treatment provides an important background for prior therapy. Understanding post-surgical recurrence patterns can help optimize the timing of initiating RET inhibitor therapy, thereby avoiding overtreatment or delays in treatment (29–31).

This study has several limitations that should be taken into account when interpreting the results. First, due to constraints in the reporting of the original RCTs, we were unable to conduct a subgroup network meta-analysis based on *RET* mutation subtypes—for example, comparing the M918T mutation versus other mutations or non-mutated types. It is well known that the M918T mutation accounts for more than 50% of sporadic MTC and exhibits greater sensitivity to *RET* inhibitors; differences in drug sensitivity among various mutation types could introduce potential clinical heterogeneity (32, 33). This population heterogeneity based on *RET* mutation status is particularly important for comparisons involving “*RET*-targeted” therapies, as *RET* inhibitors may have limited efficacy in patients with wild-type *RET*, which represents a major source of heterogeneity when extrapolating the findings of this study. Second, this study primarily focused on PFS and ORR; data on overall survival (OS) remain immature. Since most trials allowed patients in the control group to cross over to the experimental treatment after disease progression, analyzing OS benefits becomes complicated and may lead to an underestimation of the true survival advantage offered by the experimental drugs. Finally, health economic factors were not included in the evaluation. Although selpercatinib demonstrates remarkable efficacy, its high treatment costs could limit its accessibility in resource-limited settings. Future pharmacoeconomic studies will be crucial for informing global health policy development (34, 35). Although the Peters’ regression test indicated statistically significant funnel plot asymmetry (*P* = 0.009), a deeper analysis within the specific context of this study suggests that this signal should rather be interpreted as a reflection of genuine heterogeneity in treatment effects across different drug classes, rather than traditional publication bias. First, the transparency assessment revealed that all included trials had undergone prospective registration, and even the trial with the smallest sample size and negative results—EORTC-1209, involving nintedanib—had been formally published, directly refuting the hypothesis that negative findings were being concealed. Second, the observed “small-study effect” essentially stems from the graded differences in drug efficacy across the network: large, pivotal, registered trials (such as ZETA and LIBRETTO-531) primarily evaluated SRIs with breakthrough efficacy, whereas earlier or smaller trials tended to focus on conventional MKIs with relatively modest efficacy (36). This distribution pattern—where larger samples correspond to higher efficacy and smaller samples correspond to lower efficacy—is bound to manifest as funnel plot asymmetry in statistical models; yet, this very asymmetry confirms the superiority of novel therapies over older ones, rather than indicating systematic bias in data reporting. Moreover, it is important to acknowledge that, due to the limited number of included studies (n = 5), the statistical power of the quantitative analyses was low (<20%), making the results highly susceptible to influence by individual extreme values. Therefore, considering the overall symmetry observed in visual assessments, the completeness of trial registrations, and the biological plausibility of the drug mechanisms, we have good reason to believe that the conclusions of this study are not substantially affected by publication bias and thus exhibit high robustness. Finally, due to the limited number of included studies, we were unable to conduct a restricted network meta-analysis focusing solely on “first-line treatment patients with *RET* mutations only.” This represents a major limitation of our study. Ideally, a transitivity assessment would require direct comparisons among studies with identical clinical characteristics—namely, all being first-line treatments and all being *RET*-mutation positive—or robust indirect comparisons via common comparators. However, the number of eligible RCTs currently published is extremely limited: Only the LIBRETTO-531 trial fully meets the criteria of “first-line plus *RET* mutation,” while other key control studies, such as EXAM and ZETA, primarily enrolled previously treated patients or mixed mutation populations. Given the scarcity of available RCT nodes, forcing stratification by line of therapy and mutation status would result in an extremely sparse network, causing the statistical model to fail to converge or to generate excessively wide confidence intervals, thereby undermining its inferential value. Therefore, this study adopted a weighted anchor analysis using data from the entire population. This means that the conclusions of our study rely, to some extent, on extrapolating findings from the highly selected *RET*-mutation population (from the LIBRETTO-531 trial) to a broader population that includes mixed mutation types. In the broader context of multi-line therapy, although this extrapolation is biologically plausible—given that *RET* inhibitors exhibit mechanism-specific activity against *RET*-driven tumors—readers should interpret the generalizability of the results with caution. Future studies urgently need more head-to-head trials specifically targeting particular subgroups, or access to individual patient data (IPD) for more precise subgroup validation. Until then, the findings of this study should be regarded primarily as strong evidence for patients with advanced MTC harboring *RET* mutations; caution should be exercised when extending these findings to all patients with advanced MTC. We further acknowledge a critical limitation regarding patient population heterogeneity. LIBRETTO-531 enrolled only treatment-naïve RET-mutant advanced MTC patients, while the MKI trials included mixed populations with varying prior therapies and lower or unselected RET mutation rates. This difference may underestimate the comparative efficacy of MKIs. Despite our bias-mitigation strategies and sensitivity analyses verifying robustness, our conclusions are most reliable for first-line RET-mutant patients and should be used cautiously in other populations.

In addition, this study did not include the pivotal single-arm ARROW trial evaluating another highly selective *RET* inhibitor, pralsetinib (8). The primary reason for excluding the ARROW trial is that it was designed as a single-arm study lacking randomization and control, and direct inclusion would undermine the transitivity assumption underlying the network meta-analysis based on RCTs. Nevertheless, the magnitude of efficacy and safety profile observed in the ARROW trial were highly consistent with the results we derived for selpercatinib. This provides strong external validation for the conclusion that “SRIs, as a class of drugs, significantly outperform traditional MKIs.” Although subtle differences between the two remain to be further clarified by future head-to-head studies. Despite these limitations, this is the first network meta-analysis to synthesize evidence from RCTs to directly compare the efficacy and safety of SRIs with MKIs in a unified framework.

Although SRIs have shown remarkable therapeutic efficacy, acquired resistance remains an inevitable clinical challenge and the primary bottleneck limiting their long-term effectiveness (37). Recent molecular biology studies have revealed that solvent-front mutations in *RET* (such as G810R, G810S, and G810C) represent one of the major mechanisms underlying resistance to selpercatinib and pralsetinib (38, 39). These mutations occur in the solvent-front region of the *RET* kinase structure, physically obstructing the binding of the drug to the ATP-binding pocket and thereby rendering the drug ineffective. *In vitro* studies have demonstrated that the G810R mutation confers high-level resistance to selpercatinib, while some second-generation MKIs or next-generation, highly selective inhibitors (such as LOXO-260) may still remain sensitive to this mutation (20). This finding suggests that future treatment strategies might need to involve dynamic monitoring of circulating tumor DNA (ctDNA) to identify resistance mutations at an early stage and promptly adjust treatment regimens according to the specific mutation type (40). Moreover, exploring combination therapies that pair selective inhibitors with inhibitors targeting other pathways—such as SHP2 inhibitors or MEK inhibitors—could represent a promising approach for overcoming or delaying the emergence of resistance (41).

## 5 Conclusion

In summary, this network meta-analysis demonstrates that selpercatinib exhibits superior efficacy in terms of PFS and ORR, coupled with a more favorable safety profile compared to MKIs for advanced MTC. While these results suggest that selpercatinib may represent a highly effective treatment option for patients with advanced RET-mutant MTC, we acknowledge the limitation regarding generalizability due to the heterogeneity of included populations (specifically, the mix of RET mutation statuses and lines of therapy across the trials). Therefore, although selpercatinib shows a compelling benefit-risk profile, clinical decisions should be tailored cautiously, taking into account individual patient characteristics, and future head-to-head trials or individual patient data (IPD) meta-analyses focusing on homogeneously defined populations (e.g., first-line RET-mutant only) are warranted to confirm these findings.

## Data Availability

The original contributions presented in the study are included in the article/**Supplementary Material**. Further inquiries can be directed to the corresponding author.

## Supplementary material

Supplementary Figure 1 Design-by-treatment interaction model for consistency assessment. Consistency was evaluated using the design-by-treatment interaction model for PFS, ORR, and ≥Grade 3 AEs. **(A, C, E)** compare direct evidence (blue squares; LIBRETTO-531) against indirect evidence (orange squares). **(B, D, F)** display the inconsistency factor (IF; ratio of direct to indirect effects). **(A, B)** PFS: Direct (HR 0.280) and indirect (HR= 0.278) estimates aligned closely (IF= 1.007, P = 0.9870). **(C, D)** ORR: Direct and indirect estimates were identical (IF= 1.000); however, this is a mathematical artifact of the network topology. **(E, F)** ≥Grade 3 AEs: Direct (OR 0.348) and indirect (OR= 0.121) estimates differed (IF 2.889, P = 0.2660), with high uncertainty due to single-study passthrough.

Supplementary Figure 2 Risk of bias (RoB) assessment. RoB for included RCTs (ZETA, EXAM, ALTER01031, LIBRETTO-531, EORTC-1209) was assessed using Cochrane RoB 2.0 across five domains. Green indicates “Low risk,” yellow indicates “Some concerns,” and red indicates “High risk.”

Supplementary Figure 3 GRADE summary of findings. The Grading of Recommendations Assessment, Development and Evaluation (GRADE) approach assessed the certainty of evidence for MTC treatments (Treatment vs Control). Effect estimates include HR for PFS and OR for ORR and ≥Grade 3 AEs. Certainty is rated as High, Moderate, Low, or Very low.

Supplementary Figure 4 Sensitivity analyses for PFS. Sensitivity analyses tested the robustness of PFS findings across scenarios (SA1–SA5), including the main analysis (Random-effects model), fixed-effects model, exclusion of specific trials (e.g., EORTC-1209), and different bridge analyses. Consistent direction and magnitude of effects support result robustness.

Supplementary Figure 5 Publication bias assessment for PFS. Assessment of small-study effects and publication bias for PFS using: **(A)** Comparison-adjusted funnel plot; **(B)** Standard funnel plot with Egger’s regression line; **(C)** Egger’s regression test; and **(D)** Regression-based tests (Egger, Begg, and Peters). The Peters test suggested a potential small-study effect (P = 0.009), whereas Egger’s test was non-significant (P = 0.619). Analysis is limited by the small number of studies (n = 5).
