# Supplementary figures and images for: Selpercatinib versus multi-kinase inhibitors for advanced medullary thyroid cancer: A network meta-analysis of RET-targeted therapies
Source: Front Endocrinol (Lausanne). 2026 Jun 22;17:1851074. doi: 10.3389/fendo.2026.1851074 (PMC13333457; doi:10.3389/fendo.2026.1851074)

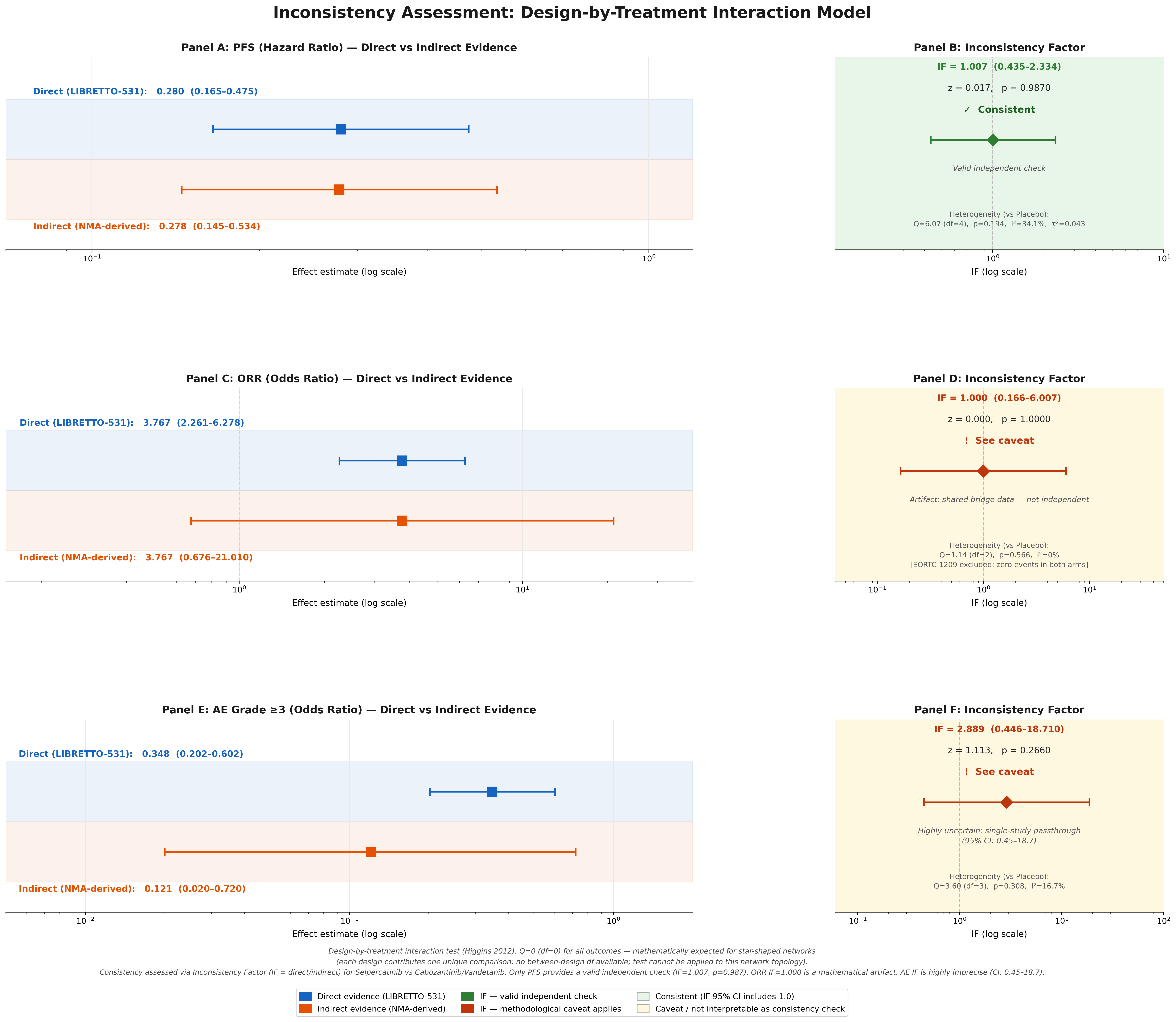

Supplement: Supplementary Figure 1 — Design-by-treatment interaction model for consistency assessment. Consistency was evaluated using the design-by-treatment interaction model for PFS, ORR, and ≥Grade 3 AEs. (A, C, E) compare direct evidence (blue squares; LIBRETTO-531) against indirect evidence (orange squares). (B, D, F) display the inconsistency factor (IF; ratio of direct to indirect effects). (A, B) PFS: Direct (HR 0.280) and indirect (HR= 0.278) estimates aligned closely (IF= 1.007, P = 0.9870). (C, D) ORR: Direct and indirect estimates were identical (IF= 1.000); however, this is a mathematical artifact of the network topology. (E, F) ≥Grade 3 AEs: Direct (OR 0.348) and indirect (OR= 0.121) estimates differed (IF 2.889, P = 0.2660), with high uncertainty due to single-study passthrough. [file Image1.tif]

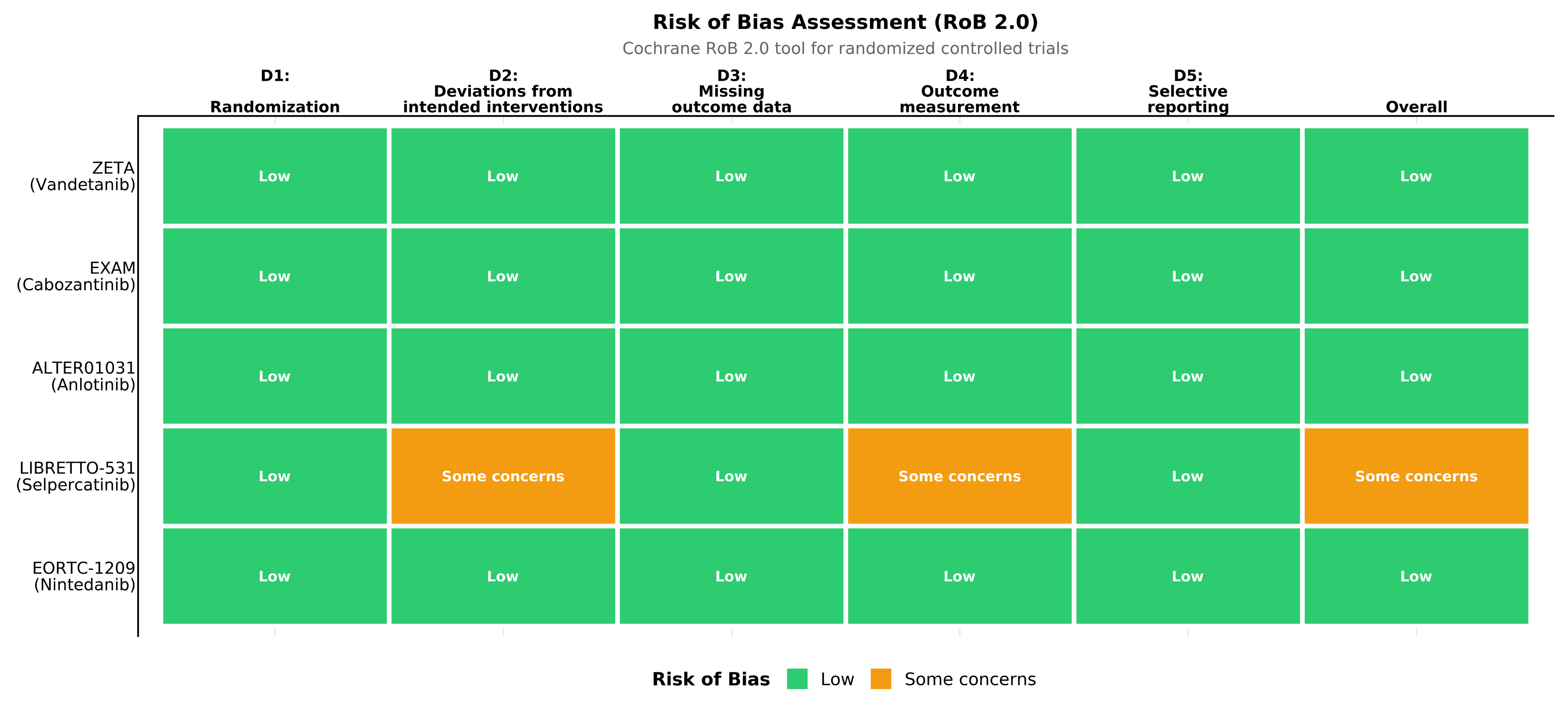

Supplement: Supplementary Figure 2 — Risk of bias (RoB) assessment. RoB for included RCTs (ZETA, EXAM, ALTER01031, LIBRETTO-531, EORTC-1209) was assessed using Cochrane RoB 2.0 across five domains. Green indicates “Low risk,” yellow indicates “Some concerns,” and red indicates “High risk.” [file Image2.tif]

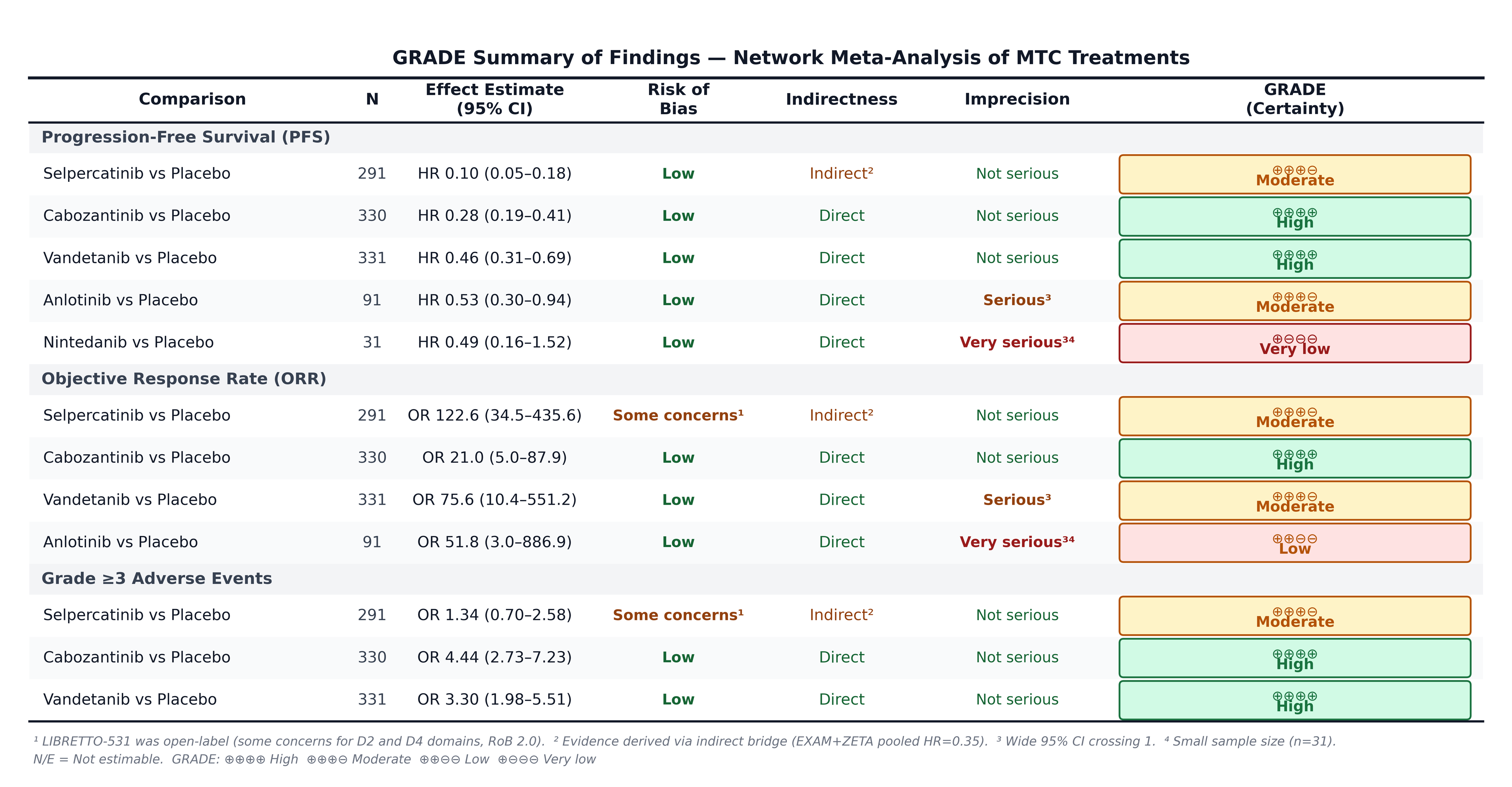

Supplement: Supplementary Figure 3 — GRADE summary of findings. The Grading of Recommendations Assessment, Development and Evaluation (GRADE) approach assessed the certainty of evidence for MTC treatments (Treatment vs Control). Effect estimates include HR for PFS and OR for ORR and ≥Grade 3 AEs. Certainty is rated as High, Moderate, Low, or Very low. [file Image3.tif]

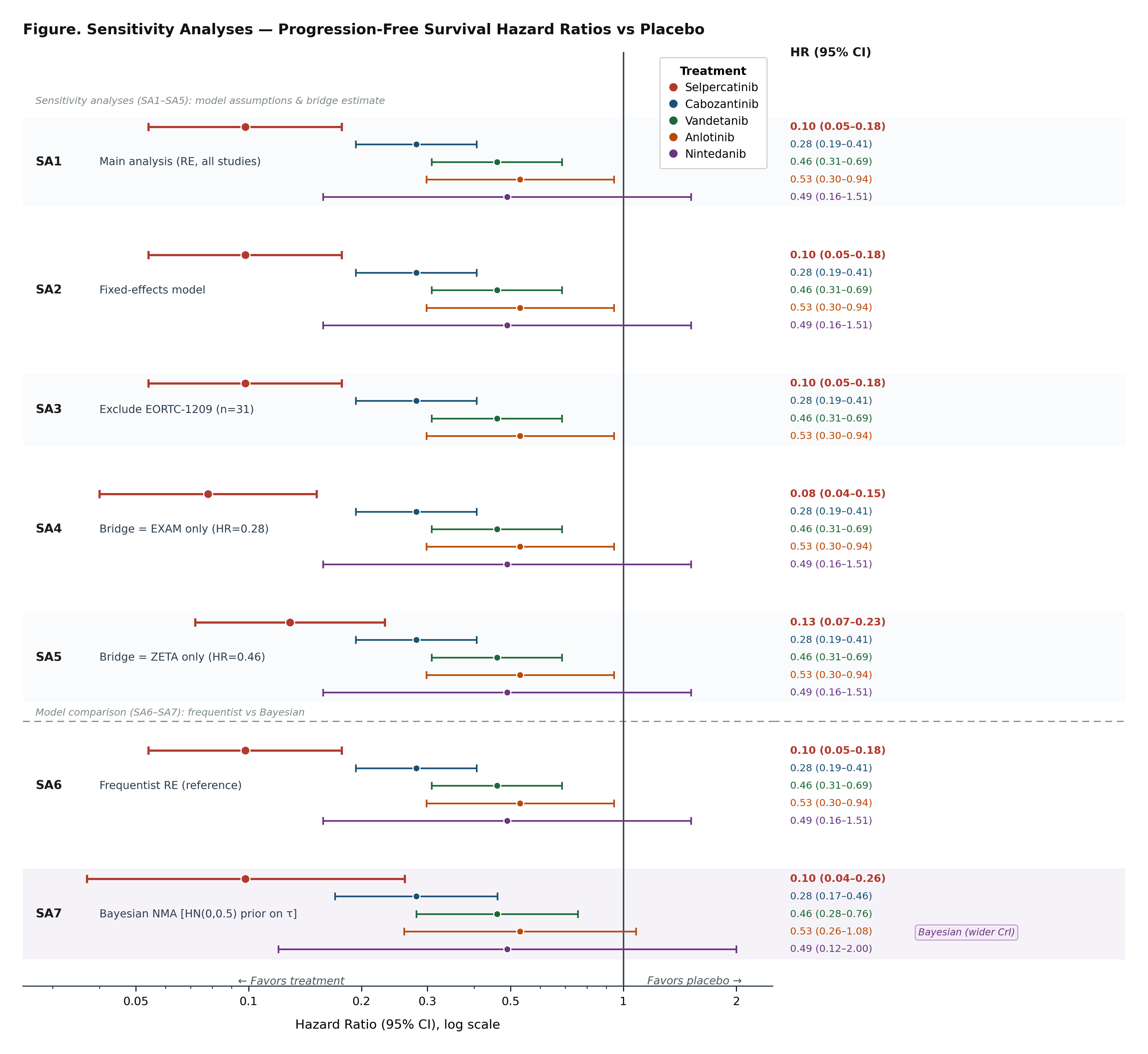

Supplement: Supplementary Figure 4 — Sensitivity analyses for PFS. Sensitivity analyses tested the robustness of PFS findings across scenarios (SA1–SA5), including the main analysis (Random-effects model), fixed-effects model, exclusion of specific trials (e.g., EORTC-1209), and different bridge analyses. Consistent direction and magnitude of effects support result robustness. [file Image4.tif]

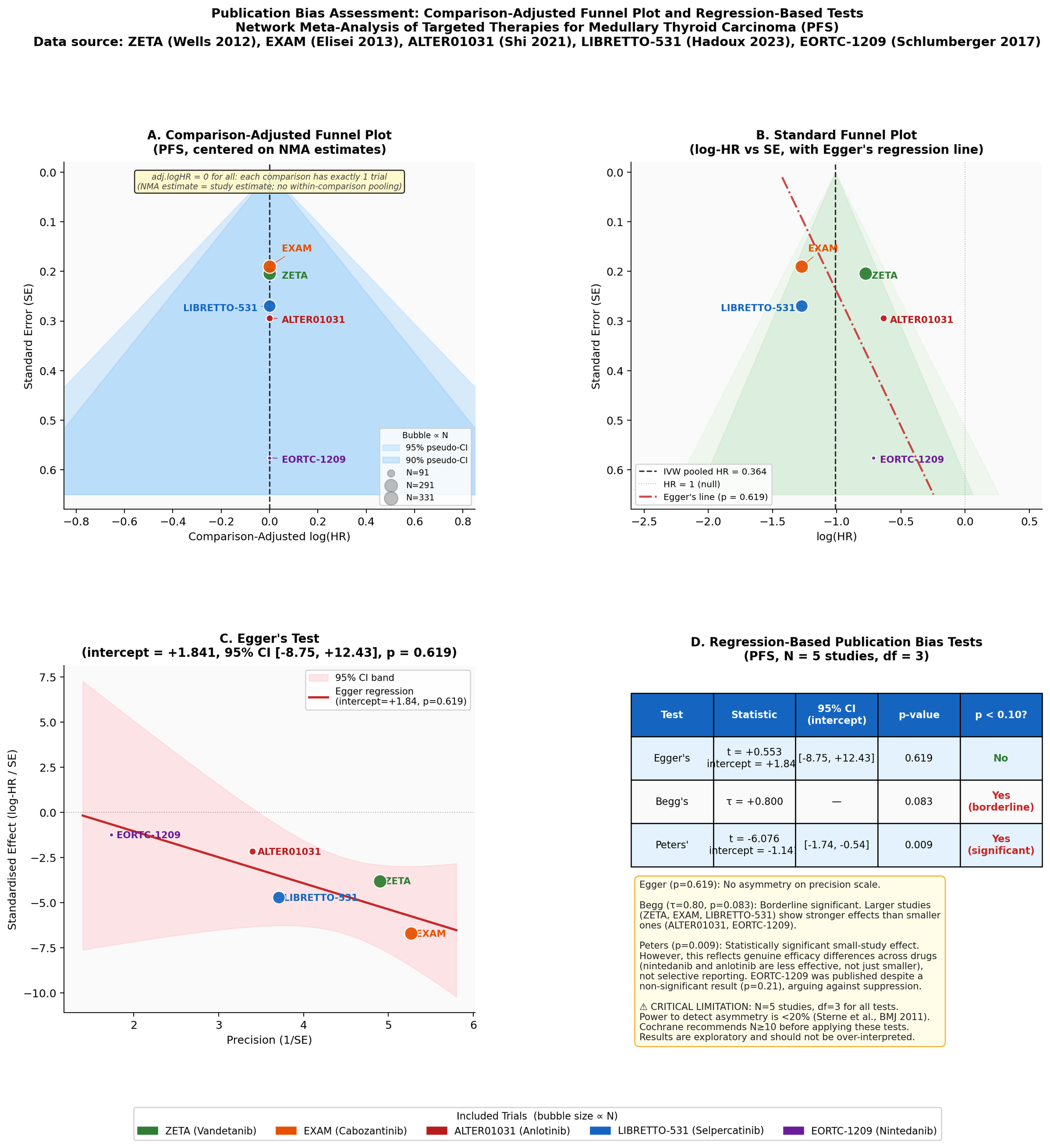

Supplement: Supplementary Figure 5 — Publication bias assessment for PFS. Assessment of small-study effects and publication bias for PFS using: (A) Comparison-adjusted funnel plot; (B) Standard funnel plot with Egger’s regression line; (C) Egger’s regression test; and (D) Regression-based tests (Egger, Begg, and Peters). The Peters test suggested a potential small-study effect (P = 0.009), whereas Egger’s test was non-significant (P = 0.619). Analysis is limited by the small number of studies (n = 5). [file Image5.tif]
